# A Comprehensive View on the Host Factors and Viral Proteins Associated With Porcine Epidemic Diarrhea Virus Infection

**DOI:** 10.3389/fmicb.2021.762358

**Published:** 2021-12-07

**Authors:** Yi Hu, Xiaohong Xie, Lingchen Yang, Aibing Wang

**Affiliations:** ^1^Laboratory of Animal Disease Prevention and Control and Animal Model, Hunan Provincial Key Laboratory of Protein Engineering in Animal Vaccines, College of Veterinary Medicine, Hunan Agricultural University, Changsha, China; ^2^Hunan Engineering Research Center of Livestock and Poultry Health Care, Colleges of Veterinary Medicine, Hunan Agricultural University, Changsha, China; ^3^PCB Biotechnology, LLC, Rockville, MD, United States

**Keywords:** porcine epidemic diarrhea virus (PEDV), host factors, viral proteins, viral infection, interaction

## Abstract

Porcine epidemic diarrhea virus (PEDV), a coronavirus pathogen of the pig intestinal tract, can cause fatal watery diarrhea in piglets, thereby causing huge economic losses to swine industries around the world. The pathogenesis of PEDV has intensively been studied; however, the viral proteins of PEDV and the host factors in target cells, as well as their interactions, which are the foundation of the molecular mechanisms of viral infection, remain to be summarized and updated. PEDV has multiple important structural and functional proteins, which play various roles in the process of virus infection. Among them, the S and N proteins play vital roles in biological processes related to PEDV survival *via* interacting with the host cell proteins. Meanwhile, a number of host factors including receptors are required for the infection of PEDV *via* interacting with the viral proteins, thereby affecting the reproduction of PEDV and contributing to its life cycle. In this review, we provide an updated understanding of viral proteins and host factors, as well as their interactions in terms of PEDV infection. Additionally, the effects of cellular factors, events, and signaling pathways on PEDV infection are also discussed. Thus, these comprehensive and profound insights should facilitate for the further investigations, control, and prevention of PEDV infection.

## Introduction

Porcine epidemic diarrhea (PED) is an acute and highly contagious intestinal infectious disease caused by porcine epidemic diarrhea virus (PEDV) (Sergeev, 2009). PEDV can cause morbidity in all age groups of pigs, with that in piglets being the highest. Clinically, it is characterized by vomiting, diarrhea, and dehydration of piglets, with a mortality rate even reaching to 100% (Hou et al., 2007). During the devastating 2013–2015 PEDV epidemics, the United States pig industry suffered serious economic losses, with a loss of approximately 7 million pigs (Antas and Woźniakowski, 2019). Due to strict biosecurity measures and feeding, the prevalence of PEDV in North America exhibited a declined trend, while the outbreaks of PEDV in Asia presented highly complex variability mainly due to the continuous occurrence and emergence of recombination or new isolates in recent years (Sun et al., 2019).

As a member of the Nidovirales order and Coronaviridae family, PEDV has a typically corolla-shaped, mostly spherical morphology, with a diameter ranging from 95 to 190 nm (including spikes) and an average of 130 nm. The sequences located at the 3′ side of the PEDV genome encode four structural proteins, namely, the spike protein (S, 150–220 kDa), membrane protein (M, 20–30 kDa), envelop protein (E, 7 kDa), and nucleocapsid protein (N, 58 kDa) (Kocherhans et al., 2001).

Similar to other coronaviruses (CoVs) (Weiss and Navas-Martin, 2005), the infection or replication processes of PEDV consist of several major steps, such as attachment and entry, viral replication enzyme translation, genome transcription and replication, structural protein translation, and virion assembly and release. Besides viral protein, viruses can interact/hijack various host factors to accomplish these processes. Therefore, a deeper understanding of these viral proteins, host factors, and their interactions will facilitate the elucidation of the pathogenic mechanisms of PEDV and accelerate the developmental pace of drugs or vaccines against PEDV. In this review, we firstly characterized PEDV viral proteins and host factors documented in the literatures, followed by the discussion of cellular events or signaling pathways involved during the viral pathogenesis, as well as the interactions among them; finally, we suggest the directions for future efforts.

## The Viral Proteins Involved in Porcine Epidemic Diarrhea Virus Infection

### 5′UTR and 3′UTR

**FIGURE 1:**
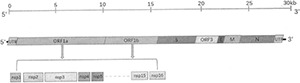
Genomic structure of PEDV. PEDV is an enveloped virus with a single-stranded positive-sense RNA genome of 28 kb in length. 5′UTR and 3′UTR are indicated as shortened gray boxes; ORF1a and ORF1b encode replicase 1a and 1b, respectively, which are the two polyprotein precursors of 16 non-structural proteins. The genes encoding four structural proteins spike (S), envelope (E), membrane (M), and nucleocapsid (N) are shown as orange, red, green, and dark blue boxes, respectively. The gene encoding ORF3 is shown as a yellow box.

Coronaviruses’ 5′UTRs vary in length, ranging from 209 to 528 nucleotides. The 5′UTR of coronaviruses has a short ORF starting with AUG at a similar location that encodes 3–11 amino acids (Morris and Geballe, 2000). The role of this ORF is unclear, while many scholars believe that it may regulate viral replication by enhancing the translation of downstream ORF (Senanayake and Brian, 1999). The 5′UTR of PEDV contains 296 nucleotides, which encodes 10 amino acids (Chen et al., 2012). The 3′UTR of coronaviruses contains 288–506 nucleotides with a poly A tail at its C terminal, and GGAAGAGC sequences at 73–80 nucleotides upstream of the poly A tail (Goebel et al., 2007; Züst et al., 2008). The 3′UTR length of PEDV is 334 bp; it is the first binding site of replicase and participates in regulating the replication process of PEDV (Chen et al., 2012). The genome of PEDV and its main genes or proteins are indicated in Figure 1.

### The S Protein of Porcine Epidemic Diarrhea Virus

This spike (S) protein is a type I glycoprotein, and three S proteins of PEDV forming a rod-shaped functional spike trimer located in the outermost layer of virus particles are the foundation for its multifunction, such as the binding of PEDV to other molecules and the entry of virus into target cells (Liu et al., 2015; Fu et al., 2017). Based on the homology analysis of its counterparts of other coronaviruses, the S protein of PEDV can be divided into two parts: S1 (1–789 aa) and S2 (790–1383 aa) (Li, 2012, 2015; Wrapp and McLellan, 2019). Previous studies have confirmed that the interaction of the S protein with the target cell surface receptor(s) is mediated by the S1 N-terminal domain (NTD) (Liu et al., 2015; Deng et al., 2016). Besides this, the involvement of the S protein in trypsin-dependent PEDV propagation in cultured cells has been suggested (Li et al., 2016). This effect mediated by proteolytic activation of trypsin or other proteases when the S protein binds to its receptor(s) (Wicht et al., 2014; Liu C. et al., 2016) is necessary for the membrane fusion, formation of syncytium, and cell entry of the virus, thereby enhancing the infectivity of PEDV (Li et al., 2015; Kim et al., 2017). This is also supported by the S protein mutations influencing its proteolytic cleave (Park J. E. et al., 2011). In addition, co-infection of PEDV variants with 214 amino acid deletion in the S protein and PEDVs with an intact S protein in the lungs of naturally infected pigs has been found recently, thereby suggesting that PEDV targets epithelial cells and submucosal glands of the airway tract, epithelial cells of the bile duct, and monocytes/macrophages (Van Diep et al., 2020). It becomes evident that the receptor binding capability and the role in viral entry allow the S protein to determine PEDV invasion and release, tissue tropism, host range, and cross-species transmission, even to affect trypsin-dependent PEDV proliferation (Wicht et al., 2014; Li et al., 2015; Liu C. et al., 2016). These findings demonstrate the multiple roles of the S protein in the PEDV infection, which should be the research emphasis on the relationship of this viral protein’s structure and functions.

### The M Protein of Porcine Epidemic Diarrhea Virus

The M protein encoded by PEDV is an essential membrane glycoprotein in the envelope. Growing evidence indicates that the M protein has multiple functions. Firstly, it can be utilized as an important antigen for developing diagnostic reagents. Indirect enzyme-linked immunosorbent assay (ELISA) using the recombinant PEDV M protein as antigen displays high sensitivity and specificity in detecting the PEDV antibody (Fan et al., 2015). Secondly, the M protein of PEDV, combined with other viral proteins, participates in the process of viral replication. For instance, the M protein, along with the N protein and other membrane proteins, takes part in viral particle assembly. Likewise, this protein, together with the E protein, participates in viral envelope assembly. Importantly, the PEDV S, E, and truncated ORF3 proteins all are confirmed to be immunoprecipitated by the M protein, indicating an interaction with the latter. In particular, CoV-related studies have revealed that the M protein interacts with the E and S proteins to participate in the CoV envelope assembly process (Nguyen and Hogue, 1997). Similarly, the interaction between the PEDV M and ORF3 proteins has suggested that these two proteins are also involved in the assembly and budding processes of PEDV (Vennema et al., 1991; de Haan et al., 1998; Wang et al., 2020). Completely dissecting the interplay of the M with other proteins may benefit the development of effective drugs for the control of this virus.

### The N Protein of Porcine Epidemic Diarrhea Virus

The N protein of PEDV is the only phosphorylated nucleocapsid protein among the known structural proteins of CoVs. As a major structural protein of PEDV, the mRNA and protein contents of the N reach to the highest during the process of viral infection. The multifunctional property of the N protein manifests in several aspects: (1) its being bound by viral RNA provides a structural basis for the helix nucleocapsid, while the resultant product is recognized by the M protein through their interaction, and then packaged into viral particles to form the core of CoVs (Shi D. et al., 2017); (2) as an alkaline phosphoprotein, the N protein is also associated with virus replication and transcription (Tan et al., 2006); (3) it participates in the biological processes related to PEDV survival (Shi D. et al., 2017; Kong et al., 2019); and (4) its localization in the nucleus may be linked to the regulation of the host cell cycle, thereby promoting the replication of PEDV (Xu et al., 2013b). Similar to the M protein, the N protein executes various functions, while its action mechanisms in the latter three aspects have not been fully elucidated.

### The E Protein of Porcine Epidemic Diarrhea Virus

The E protein is a small membrane glycoprotein, which is distributed on the surface of the virus envelope. According to the primary and secondary structure analysis of this protein, it can be divided into three parts: the short amino terminal hydrophilic region, the α helix structure with a length of about 25 aa containing the transmembrane region, and the long carboxyl terminal region (Torres et al., 2007). So far, the functions of the E protein have not been extensively studied. A previous study indicated that the E protein could induce endoplasmic reticulum stress and upregulate the expression of IL-8 and Bcl-2 through activating the NF-κB pathway in host cells, suggesting that it might be associated with the inflammatory response and the persistence of PEDV infection (Xu et al., 2013a). Similarly, a recent study showed that the E protein might facilitate to evade host innate immunity through suppressing the RIG-I-mediated signaling (Zheng et al., 2021). These findings suggest a role of the E protein in triggering or affecting host immune response. Additionally, multiple functions for the CoV E protein have been suggested or confirmed (Ruch and Machamer, 2012); however, these are not validated for the PEDV E protein. Therefore, an in-depth investigation on the PEDV E protein is required.

### The ORF3 of Porcine Epidemic Diarrhea Virus

As a unique feature to other coronaviruses such as TGEV and SeCoV, the sequence located between the PEDV S and E genes is responsible for encoding the non-structural ORF3 protein. It contains four transmembrane regions and forms a homologous tetramer structure (Park S. J. et al., 2011; Chen et al., 2015). As an accessory protein, the ORF3 is generally believed not to play a role in the reproduction process of PEDV. However, a series of investigations on ORF3 reveal that this may not be the case. Firstly, bioinformatic analysis demonstrated that the complete ORF3 protein can form ion channels. Moreover, silencing the ORF3 gene would lead to a decrease of virus titer, thereby suggesting that it might play a regulatory role in the process of PEDV infection (Wang et al., 2012). Secondly, the ORF3 gene of PEDV was confirmed to be able to promote the formation of the vesicle structure, prolong the S phase of target cells, and enhance the proliferation of the attenuated strain expressing a truncated ORF3, further indicating its implication in the replication process of PEDV (Ye et al., 2015). Additionally, the interaction of the PEDV ORF3 with the cellular VPS36 was found to inhibit virus replication (Kaewborisuth et al., 2019). Furthermore, ORF3 was observed to inhibit the production of IL-6 and IL-8 through affecting NF-κB signaling, thereby promoting viruses to escape from host innate immunity (Wu et al., 2020). Altogether, these studies highlight the important roles of the ORF3 gene/protein in the PEDV infection. In spite of these, further investigation on the role of the ORF3 accessory protein, especially in terms of host–virus interaction, is demanded.

### The Non-structural Proteins of Porcine Epidemic Diarrhea Virus

The translated products of poly-proteins pp1a and pp1ab from the ORF1a and ORF1b transcripts are cleaved into 16 mature replicase proteins nsp1-nsp16 (Snijder et al., 2003; Thiel et al., 2003). A previous study indicated that the nsp1 protein has the most pronounced effect on the host innate immune response among all encoded proteins of PEDV (Zhang et al., 2016). Moreover, nsp1 is regarded as an essential virulence determinant and a target for vaccine development since it can disrupt host gene expression and stimulate antiviral response, thereby blunting the innate immune response of the host to the coronavirus pathogens. This notion was supported by several facts that as an effective IFN antagonist, nsp1 was found to interfere with the IRF- and NF-κB-mediated induction of type I and type III IFNs (Zhang et al., 2017, 2018), and as an inhibitor, it was demonstrated to regulate the biological functions related to host proliferation and immune escape, thus creating a favorable environment for viruses (Shen et al., 2020). Further studies on the structure and biochemical characteristics of the PEDV nsp1 revealed that the residues 67∼71, 78∼85, and 103∼110 of it constituting functional regions are responsible for the inhibition of host protein synthesis (Narayanan et al., 2015; Shen et al., 2018, 2019). Similarly, other nsp molecules of PEDV were confirmed to play a role in regulating immune response, thus affecting viral infection. For instance, nsp2 was found to target FBXW7 degradation through their interaction, thus bypassing the innate immune response of the host and promoting the replication of PEDV (Li et al., 2021). The nsp3-mediated de-ubiquitination of RIG-I and STING resulted in downstream signal interruption and type I IFN expression inhibition (Liu Y. et al., 2016). nsp4 could induce the expression of pro-inflammatory cytokines and chemokines such as IL-1α, IL-1β, TNF-α, CCL2, CCL5, and CXCL8 *via* targeting the NF-κB pathway during the viral infection (Yu et al., 2019). The nsp5 protein, also called 3CLpro, was observed to split proproteins between nsp5 and nsp16, thus turning them into mature proteins which participate in various stages of virus replication (Tomar et al., 2015). Moreover, the functional experiments on nsp5 also revealed that it plays an antagonistic role of interferon through shearing Gln231 in the NEMO (NF-κB essential modulator) protein (Wang et al., 2016). Interestingly, the PLpro domain of nsp3 and the 3CLpro domain of nsp5 were validated to have a viral replication-promoting effect, which is related to its function of processing pp1a and pp1ab polyprotein precursors into nsps (Richard and Tulasne, 2012). Recent evidence indicated that nsp6 induces autophagy through the PI3K/Akt/mTOR signaling pathway to promote PEDV replication (Lin et al., 2020). The vital role of nsp9 in viral replication has also been suggested by several studies (Sutton et al., 2004). Current evidence revealed that nsp12 is an RNA-dependent RNA polymerase and nsp13 is a helicase (Xu et al., 2011; Ren et al., 2021); therefore, their effect on viral replication can be expected. Although both nsp14 and nsp16 have been considered as the antagonists of innate immunity, the latter was a more effective modulator of immune-related genes (Shi et al., 2019). Moreover, nsp16 relied on the KDKE tetrad, which can effectively reduce PEDV-induced IFN-β production and promote virus proliferation (Shi et al., 2019). Intriguingly, nsp10 was found to enhance the inhibitory effect of nsp16 on IFN-β production (Shi et al., 2019). Additionally, nsp15 and nsp16 were also verified to be effective interferon antagonists, since inactivation of them and nsp1 produced a highly attenuated virus which does not cause diarrhea in pigs and induces a neutralizing antibody response in virus-infected animals (Deng et al., 2020). Among all the 16 nsps of PEDV, nsp1, nsp3, nsp7, nsp14, nsp15, and nsp16 could inhibit IFN-β and IRF3 promoter activity (Zhang et al., 2016), while nsp1, nsp3, nsp5, nsp8, nsp14, nsp15, and nsp16 were found to suppress the activity of type III IFN, which is mediated by the IRF1 signaling pathway (Zhang et al., 2018). Collectively, the available studies demonstrate the importance of the PEDV non-structural proteins in terms of viral infection; thus, it is reasonable to speculate that these proteins may promote the effect of vaccines and the signaling pathways affected by them can be targets for the development of druggable agents, if related issues are further addressed.

## Host Factors Involved in the Porcine Epidemic Diarrhea Virus Infection

It is well known that viral infection is a multistep process including adsorption, cell entry, dehulling, biosynthesis, assembly, and release. Besides the viral proteins of PEDV, the implication of numerous host factors including receptors in these processes has been confirmed, which is described as the following in accordance with the infection stages.

### Attachment and Entry

Adsorption of viruses to host cells is the first and decisive step for determining the virus tropism, namely, the ability of different viruses to infect various cell types. Correspondingly, virus-specific receptors on the host cell surface are strongly related to the tissue tropism and host range and mediate the entrance of viruses including PEDV into host cells. In this regard, the S protein of PEDV plays a major role in this process by interacting with the corresponding host factors, thereby achieving the adsorption and entry into target cells (Li et al., 2016; Li C. et al., 2017).

### Porcine Aminopeptidase N

Porcine aminopeptidase N (pAPN) is widely distributed in the small intestine and kidney of pigs (Li et al., 2007). The functions of pAPN in the pathogenic mechanisms of PEDV infection have extensively been studied in recent years. Although the conclusion of the receptor role for pAPN in PEDV infection remains elusive, this view is indeed supported by partial facts. pAPN was able to bind to the S1 region of the PEDV S protein (Li et al., 2007), while ST cells efficiently expressing exogenous pAPN facilitated the PEDV infection and proliferation; furthermore, this action was closely associated with the distributed density of pAPN (Nam and Lee, 2010). However, other studies indicated that the presence of pAPN does not render Vero cells susceptible to PEDV infection (Oh et al., 2003) and the capability of pAPN to enhance the infectivity of PEDV is related to its aminopeptidase activity rather than its receptor role (Shirato et al., 2016). Whether this discrepancy is only due to various cell lines used in these studies, because the results obtained in ST cells cannot be replicated in HeLa cells, or whether the existence of other routes of PEDV infection results in variable consequences, remains to be further classified.

### Sialic Acid

It is recognized that a large number of the main receptors for many viruses belong to glycoprotein or glycolipid, with sialic acid or sialic acid derivatives at the end (Sun et al., 2016). In this regard, the ability of PEDV binding to sialic acid, such as carbohydrates (Neu5Ac) and proteins especially glycoproteins or glycolipid molecules located at the cell surface, has also been confirmed in previous studies (Künkel and Herrler, 1993; Peng et al., 2012). Moreover, using glycoproteins as receptors is an important strategy for viral intestinal pathogenicity because the properties of glycoproteins facilitate viral binding to mucin on the surface of epithelial cells in animal viscera (Wang, 2002). The effort of Wrapp et al. to parse the perfusion conformation of the PEDV S protein using cryo-electron microscopy at a resolution of 3.1 Å revealed the presence of the sialic acid-binding domain at the N terminal of the S1 subunit (Wrapp and McLellan, 2019). The recognition of sugars as co-receptors for PEDV seems to be a strategy for adapting organisms to this class of diarrhea-causing viruses (Isa et al., 2006), suggesting that the binding of PEDV to sialic acid may favor to survive in adverse intestinal conditions (Deng et al., 2016). These observations suggest that sialic acid is an adsorption factor that promotes PEDV infection and provides a basis for understanding the molecular mechanisms that drive the earliest stages of PEDV infection.

### Heparan Sulfate

Heparan sulfate (HS) is known for its role as an attachment factor by many viruses to enter cells (Barth et al., 2003). Its similar function in mediating the attachment/absorption of PEDV to its host cells has been suggested (Huan et al., 2015), as confirmed by a series of observations. First, HS is a complex polysaccharide located on the cell surface and extracellular matrix (Sarrazin et al., 2011). Second, glycosaminoglycans (GAGs), composed of several covalently attached HS chains, can provide sites for the binding of various viruses to eukaryotic cells (Barth et al., 2003). Third, it has been demonstrated that PEDV utilizes HS to attach to Vero cells, while pretreatment with heparan can inhibit PEDV infection. Moreover, both the N- and O-linked sulfate groups within the HS carbohydrate structure are functionally important for the binding of PEDV to target cells (Huan et al., 2015). Last, the binding ability of PEDV to Vero cells is reduced following the enzymatic removal of cell-surface HS or the inhibition of HS biosynthesis by treatment with chlorate (Huan et al., 2015). These results robustly suggest that HS is at least an adsorbing factor for the infection of PEDV in studied Vero cells.

### Epidermal Growth Factor Receptor

Epidermal growth factor receptor (EGFR) is widely distributed on the membrane surface of mammalian epithelial cells and fibroblasts (Singh and Harris, 2004). Importantly, the EGFR-mediated signaling pathway plays an important role in cell proliferation, differentiation, and apoptosis, even viral infection. In this regard, the activation of EGFR occurring in the early stage of PEDV infection has been found in a recent study. Furthermore, this EGFR activation by PEDV infection might be mediated by the direct interaction between EGFR and the S protein, which in turn enhanced PEDV infectivity. Mechanistically, the effect could be associated with the suppression of type I interferon antiviral activity following PEDV-induced EGFR-STAT3 signaling pathway activation (Yang et al., 2018), suggesting that PEDV can effectively utilize EGFR to inhibit cellular antiviral defense. Despite these, more details about these signaling-related events remain to be further elucidated.

### DC-SIGN (CD209)

DC-SIGN (also called CD209), specifically expressed on the surface of dendritic cells (DCs), is a C-type lectin-like cell-surface receptor with multiple functions. Its expression is also found in gastric and intestinal mucosa and other epithelial cells. Initially, DC-SIGN was proved only to be a pattern recognition receptor (PRR) and adhesion molecule for dendritic cells to recognize pathogenic infections and participate in the innate immunity of organisms. Accumulating evidence has indicated that DC-SIGN also acts as the receptor of many viruses for infecting hosts and the mediator of virus immune escape. Importantly, DC-SIGN, together with other PRRs, can identify and capture viruses, further swallowing and storing them to evade lysosome degradation, then participate in antigen presentation, thereby achieving the mediation of virus infection and *in vivo* dissemination (Halary et al., 2002). Studies have shown that the mannose carbohydrate residues on the surface of the coronavirus spike protein can bind to the DC-SIGN receptor and play an important role in the process of coronavirus infection (Zhang et al., 2012). Human aminopeptidase N (hAPN) has been confirmed to be a cell receptor of HCoV-229E, which is a common coronavirus of the upper respiratory tract. Furthermore, a series of experiments have also validated that HCoV-229E can use CD209L as one of its receptors (Jeffers et al., 2006). Based on the similarity of the sequence of HCoV-229E with that of PEDV, CD209L may be a receptor of PEDV as well. However, substantial evidence is required to verify this concept in the future.

### Type II Transmembrane Serine Proteases

Type II transmembrane serine proteases (TTSPs), being a family with more than 20 members, can be mainly divided into four subgroups: HAT/DESC, hepsin/TMPRSS, matriptase, and corin (Szabo and Bugge, 2008). TTSPs consist of several functional domains expressed in many tissues and cell mucosal epithelia; moreover, their localization in the respiratory mucosal epithelium often facilitates respiratory virus infection (Böttcher-Friebertshäuser et al., 2010). This role of TTSPs is closely associated with their protease activity. Intriguingly, the serine protease inhibitor ABESF-HCl could significantly inhibit the replication of PEDV in Vero cells (Park et al., 2014). Furthermore, the culture of PEDV could be achieved in transmembrane protease serine 2 (TMPRSS2), stably expressing Vero cells even in the absence of trypsin. Meanwhile, indirect immunofluorescence revealed TMPRSS2-induced cell fusion in virus-infected cells (Shirato et al., 2011). Likewise, a similar role for mosaic serine protease large-form (MSPL) in enhancing the *in vitro* proliferation of PEDV has been described (Shi W. et al., 2017). These suggest that like trypsin, the promoting effect of TMPRSS2 and MSPL on PEDV proliferation may be ascribed to their ability of catalyzing the cleavage of the S protein, thus enhancing the entry and release of viral particles during PEDV infection. Additionally, DPP4, with the activity of protease and the wide distribution in many tissues and cells, is very conserved among various species and plays an important role in the infection of MERS-CoV and other emerging human coronaviruses (Ohnuma et al., 2013). Although robust evidence remains to be provided, CRISPR/Cas9 technology-mediated ablation of CD26/DPP4 gene in target cells should help to illustrate this issue.

### Tight-Junction Proteins

Tight-junction proteins widely present between the epithelial cells and endothelial cells are responsible for closing the cell gap and preventing the free entry and exit of substances inside and outside the epithelial layer (Guttman and Finlay, 2009). Tight-junction proteins composed of transmembrane proteins and cytoplasmic proteins are complex structures formed by the interaction of various proteins. They are linked to microfilaments by cytoplasmic binding proteins, which can be divided into transmembrane proteins (e.g., occludin and claudin) and cytoplasmic proteins (e.g., ZO-1, ZO-2, and ZO-3) (González-Mariscal et al., 2008). Importantly, several tightly linked proteins including occludin, claudin, CAR, and JAM were validated to act as receptors for viruses (Mateo et al., 2015; Torres-Flores and Arias, 2015). Moreover, viruses were found to invade the epithelium by binding and destroying these tight-junction proteins. Similarly, claudin-6/9 was also identified to be an invasive co-receptor in endothelial cells (Evans et al., 2007; Ploss et al., 2009). Notably, PEDV was found to cause structural alterations in the barrier integrity both *in vitro* and *in vivo* through modulating related proteins of the tight junction and adhesion junction in the early stage of infection (Zhao et al., 2014). Furthermore, this effect of PEDV on the cell junction was achieved by affecting the MAPK pathway, since inhibition of the MAPK pathway could regulate the changes in the tight junction of cells (Zhao et al., 2014). In particular, the essential role of the tight-junction protein occludin in PEDV infection during late-entry events has been suggested and characterized (Luo et al., 2017). The tight junctional distribution of occludin was pronouncedly affected by PEDV infection. Furthermore, overexpression or downregulation of occludin promoted or reduced the susceptibility of target cells to PEDV infection, respectively (Luo et al., 2017). On the other hand, the micropinocytosis-mediated occludin internalization process was promoted by the PEDV entry; this protein might serve as a scaffold in the vicinity of PEDV entry (Luo et al., 2017). Interestingly, although PEDV and occludin are mutually influenced by each other, the evidence of their direct interaction is absent. Additionally, it remains undetermined whether other tight-junction proteins play similar or various roles in the infection of PEDV. Future works are warranted to elucidate these issues.

### Integrin

Integrin is a cell membrane receptor family composed of α subunits and β subunits. Currently, 18 α subunits and 8 β subunits have been identified. They can form 24 heterodimer molecules by non-covalent bonding (Hynes, 2002). Integrins are found in almost all plants and animals, but there are a lot of variations between different species. They are mainly involved in cell–cell and cell–extracellular matrix (ECM) interactions and mediate the process of cell proliferation, differentiation, migration, and adhesion (Moser et al., 2009). Previous studies have shown that integrins act as receptors or co-receptors for viral infection (Kotecha et al., 2017; Nestić et al., 2019). Furthermore, the presence of integrins α3, αv, β1, β3, β4, and β6 rendered Vero cells susceptible to PEDV infection (Guo et al., 2014). Importantly, a series of evidence has proved that integrin plays a role in PEDV’s entry into cells by interacting with the S protein. Firstly, overexpression of integrin αvβ3 enhanced the PEDV infection to Vero E6 cells and IECs. Secondly, inhibition of integrin αvβ3 by siRNA or anti-integrin αvβ3 antibodies or arginine–glycine–aspartate (RGD) peptides could inhibit PEDV infection. Moreover, integrin αVβ3 and pAPN were found to synergistically enhance PEDV replication. These results demonstrate that integrin αvβ3 may be a co-receptor but not a functional entry receptor of PEDV (Li et al., 2019) and also suggest that the entry of PEDV into host cells may depend on a variety of cellular receptors.

### Genome Replication and Transcription

When PEDV enters the cytoplasmic exfoliation, the RNA genome in the virion is released. Infected cells generally contain seven to nine virus-specific mRNAs, which carry the same 3′ mRNA, whereas the longest one is viral genomic RNA. The PEDV replicase synthesizes full-length negative stranded RNAs using genomic RNA as a template, and then these newly synthesized RNAs function as a template for further synthesis of new genomic RNA (Masters, 2006). Although genome replication/transcription is regarded to be mediated primarily by viral replicating enzymes, multiple host factors are also involved in this process. Moreover, the N protein of PEDV, as an RNA companion, has an important role in PEDV replication and transcription, while the interaction between host factors and the N protein plays a regulatory action.

### Nucleophosmin 1

The NPM1 (B23.1) protein, derived from the main transcription form and the longest transcript of the NPM gene, is mainly located in the granular region of the nucleolus. As a nuclear shuttle protein, NPM1 plays multiple roles in the nucleolus including centrosome duplication, ribosome biogenesis, intracellular transport, apoptosis, and mRNA splicing (Lindström, 2011). Importantly, a series of evidence has demonstrated the interaction of the PEDV N protein with the NPM1 protein, as well as their contribution to PEDV infection. Firstly, the interaction and co-localization of those two proteins have been confirmed by both immunoprecipitation (IP) and GST-pull-down assay, as well as confocal microscopy, respectively. Moreover, the 147–294 aa region of PEDV N protein and the 189–294 aa region of NPM1 were verified to be essential for their interaction. Secondly, PEDV infection was found to cause considerable upregulation of the NPM1 expression. In particular, the NPM1 overexpression could promote the expression of the N protein and the proliferation of PEDV, while its downregulation led to converse consequences. Thirdly, mechanically the interaction between the N and NPM1 proteins could prevent the cells from being cut off, increase the cell resistance to apoptosis, and avoid premature cell death, thus enhancing virus replication (Shi D. et al., 2017). Therefore, the interaction of the PEDV N protein with NPM1 and the resultant changes in host cell survival may benefit for the development of vaccines and therapeutics for pigs.

### Heterogeneous Nuclear Ribonucleoprotein A1

Heterogeneous nuclear ribonucleoprotein (hnRNP) is a general term designated for the members of the RNA binding protein family, which consists of at least twenty members. Heterogeneous nuclear ribonucleoprotein A1 (hnRNPA1), one of the most abundant members of this family (Chang et al., 2017), can be divided into two different parts: the N-terminal functional region with two closely linked RNA recognition motifs mainly responsible for binding to RNA, and the C-terminal glycine enrichment region with RNA-binding sites and localization sequence M9 principally involved in RNA binding, cell localization, and protein–protein interaction (Bekenstein and Soreq, 2013). As a multifunctional protein, hnRNPA1 widely participates in the regulation of RNA transcription, splicing, nuclear shuttle, and the translation of cellular and viral proteins. The binding of hnRNPA1 to the N proteins of other coronaviruses like MHV and SARS-CoV has been confirmed by a series of experiments (Wang and Zhang, 1999). Likewise, the binding and co-localization of hnRNPA1 with the N protein of PEDV have been verified, implying the involvement of hnRNPA1 in the formation of the PEDV replication–transcription complex (Li et al., 2018). It remains to be determined whether the same binding site of the PEDV N protein is used to interact with hnRNPA1 as those of other coronaviruses such as MHV and SARS-CoV.

### Assembly and Release

Successful PEDV replication requires the coordinated production, processing, and assembly of each protein and nucleic acid of the virus, as well as the release of progeny viruses capable of infecting new cells from infected cells. Initially, the interaction of the same type M proteins provides a scaffold for the morphogenesis of the virus, while the interaction between the M-S and M-N facilitates the recruitment of the structural components of the virus to the assembly site (Ye and Hogue, 2007). Finally, the newly generated viral particles are transported in the smooth vesicles and released by the exocytic pathway of exocytosis. In this aspect, multiple host factors also take part in these processes of coronavirus including PEDV.

### Bone Marrow Stromal Cell Antigen 2

As the first defense line against pathogenic microorganism invasion, natural immune response plays a vital antiviral role in the early stage of virus infection. The critical action of interferon (IFN) in the process of virus infection and proliferation has long been recognized. As an IFN-induced common natural immune limiting factor (Kupzig et al., 2003; Sauter, 2014), BST2 could suppress viral production by affecting the release of viruses from infected cells (Van Damme et al., 2008). By analyzing the distribution of BST2 in tissues, Kong et al. found that in spite of its expression in almost all tissues and organs, BST2 exhibited a high level in immune tissues and organs, large intestine, small intestine, and lungs, hinting a key role of BST2 in early natural immune response (Kong et al., 2019). Indeed, the BST2-overexpressing Vero cells had much less virus content than the control cells; similarly, the viral titer in the cell supernatant was also pronouncedly reduced. On the contrary, PEDV proliferation was remarkably enhanced in Vero cells when the BST2 gene level was downregulated, suggesting that the BST2 protein could restrain PEDV proliferation in Vero cells. Furthermore, this function of BST2 might be achieved by binding and degrading the N protein of PEDV (Kong et al., 2019). Accordingly, selective autophagy as a novel antiviral mechanism was suggested for the action of BST2 on PEDV replication.

### Eukaryotic Translation Initiation Factor 3 Subunit L

Eukaryotic protein synthesis consists of three stages, namely, initiation, elongation, and termination, each of which involves a different set of protein factors. This event mainly occurs in the ribosomes within the cytoplasm, with the participation of a series of eIFs. So far, 13 translation–initiation factors have been identified in eukaryotes, of which eIF3 has the largest molecular weight (∼650 kDa) and is composed of 8–13 polypeptides (Asano et al., 1997). Eukaryotic translation initiation factor 3 subunit L (eIF3L) is one of the subunits of the eukaryotic translation initiation factor eIF3. A previous study revealed that eIF3L could inhibit the replication of yellow fever virus (YFV) by binding to the viral NS5 protein (Morais et al., 2013). In particular, Wang et al. established the PEDV M protein interaction group by immunoprecipitation (Co-IP) combined with liquid chromatography–mass spectrometry (LC-MS/MS) and identified 218 kinds of host cell proteins that interact with the M protein, including eIF3L. Compared with negative controls, a reduced eIF3L expression was strongly linked to a significantly increased PEDV production, suggesting that this factor plays a negative regulatory role in viral replication (Wang et al., 2020). Therefore, identifying PEDV M interacting proteins will further contribute to addressing the process of virus replication.

### Peptidyl-Prolyl *Cis-Trans* Isomerase D and S100 Calcium-Binding Protein A11

Peptidyl-prolyl *cis-trans* isomerase D (PPID), also named as CyP40, is a member of the peptidyl-prolyl *cis-trans* isomerase (PPIase) family. It is conducive to protein folding, ligand binding, and nuclear sorting of glucocorticoid, estrogen, and progesterone receptors (Pfefferle et al., 2011). Silencing PPID had a protective effect on the UVA-induced apoptosis of human keratinocytes (Jandova et al., 2013). S100 calcium-binding protein A11 (S100A11) is a member of the EF-hand Ca2 + -binding protein S100 family. It plays a key regulatory role in a variety of cellular processes associated with cancer, including proliferation, apoptosis, cell cycle, migration, invasion, and epithelial–mesenchymal transformation (EMT) (Meng et al., 2019). Dong et al. identified 40 host cell proteins that interact with the PEDV M protein, including PPID and S100A11, using a proximity-labeling enzyme APEX2 (a chimeric soybean peroxidase). Co-immunoprecipitation (Co-IP) confirmed the interaction between the M and five proteins (e.g., RIG-I, PPID, NHE-RF1, S100A11, and CLDN4). Moreover, siRNA knockout of PPID and S100A11 genes significantly increased the virus production, indicating that the proteins encoded by these two genes interfered with or downregulated the replication of the PEDV viruses (Dong et al., 2021). This study further highlights the key role of the PEDV M protein in interacting with multiple host factors in terms of virus replication.

## Other Factors in the Host Cells Affecting Porcine Epidemic Diarrhea Virus Infection

### Activity of Cytokines and Regulation of Signaling Pathways

As the invaders, viruses should have the capability to adjust the activities of cytokines and regulate intracellular signaling pathways of the host cells after invasion, thereby facilitating the replication and proliferation of viral particles. Indeed, the occurrence of pronounced expression changes of numerous proteins was identified by proteomic analysis in PEDV-infected Vero cells. These proteins were found to participate in various biological processes such as apoptosis, signal transduction, and stress response (Zeng et al., 2015). For instance, PEDV infection could activate the components of the intracellular MAPK signaling pathway including ERK (extracellular signaling-regulated kinase), p38 MAPK, and JNK (c-Jun N-terminal kinase) (Kim and Lee, 2015). In addition, PEDV infection-induced endoplasmic reticulum (ER) stress response and the activation of NF-κB signaling have been found and described as well (Wang et al., 2012; Xu et al., 2013a). Therefore, it is reasonable and logical to believe that PEDV-triggered alterations of protein expression, cellular response, and signaling collectively create a conducive microenvironment for its proliferation in the host cells.

### Cell Autophagy

It has been recognized that autophagy is not only a lysosome-dependent degradation pathway but also a defense mechanism. Growing studies have demonstrated the fundamental functions of autophagy in the process of virus infection. On the one hand, autophagy can induce an innate immune response to suppress the proliferation of viruses; on the other hand, viruses evolve various strategies to defend against and escape the destructive effects of autophagy and even use it to promote their proliferation (Sun et al., 2014). More relevantly, TGEV-induced autophagy in the ST and PK-15 cell lines has been observed. Interestingly, silencing the three main autophagic proteins could considerably increase viral load, indicating the inhibitory role of autophagy in TGEV replication (Guo et al., 2016). The specific role of autophagy in PEDV proliferation has also been verified in a previous study. The viral titer of PEDV was considerably decreased following the inhibition of cellular autophagy with its inhibitors (i.e., 3-MA or CQ), while increased proliferation of PEDV was observed upon the induction of cellular autophagy by its inducer (e.g., rapamycin). A decreased viral titer of PEDV was similarly achieved by silencing the expression of the key autophagy genes Beclin 1 and ATG5. Although these observations robustly demonstrate the implication of cell autophagy in the replication of PEDV (Guo et al., 2017), the mechanism underlying this cellular event remains elusive.

## The Interaction of Viral Proteins and Host Factors

**FIGURE 2:**
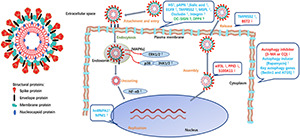
The involvement of numerous host factors and viral proteins in the different processes of PEDV infection. During the process of virus attachment and entry into cells, the host factors pAPN, HS, sialic acid, EGFR, TMPRSS2, MSPL, occludin, and integrin are found to promote PEDV infection by interacting with the S protein. DC-SIGN and DPP4 may play a role in this process as well, while more robust evidence is needed. HnRNPA1 and NPM1 are verified to interact with the N protein of PEDV, thus participating in the transcription and replication stages. Finally, TMPRSS2, BST2, eIF3L, PPID, and S100A11 are identified to take part in the assembly and release of the virus. PEDV also utilizes the p38 MAPK and JNK signaling pathways for optimal replication. Similarly, PEDV infection induces endoplasmic reticulum (ER) stress response and activation of the NF-κB signal also contributes to PEDV replication. Additionally, PEDV can facilitate its replication by affecting autophagy. ↑, ↓ in the figure denote promotion or inhibition, respectively.

As described above, intensive investigations have not only confirmed the involvement of some structural or non-structural proteins of PEDV but also verified the important contribution of some identified host factors in the forms of proteins, signaling pathways or events, or physiological processes in target cells to PEDV infection. Two major features can therefore be deduced: different viral proteins and host factors participate in various stages of PEDV infection, and the contribution of these proteins/factors is mainly achieved by their mutual interactions. To clearly illustrate these, we summarize the major functions of PEDV viral proteins and host factors in Table 1 and provide an overall view of their participation and interaction during PEDV infection, as shown in Figure 2. Briefly, at the initial stage of PEDV infection, identified host factors so far, including pAPN, sialic acid, HS, TMPRSS2, MSPL, occludin, and integrin, are confirmed to interact with the S protein, thereby facilitating the attachment and entry of the PEDV viral particles into target cells. Next, the host factors hnRNPA1 and NPM1 interact with the N protein of PEDV to promote viral transcription and replication. Subsequently, the host factor BST2 inhibits PEDV replication by binding and degrading the N protein of PEDV, and the cellular factors eIF3L, PPID, and S100A11 repress PEDV replication by binding the M protein of PEDV, while TMPRSS2 plays a role in the release of PEDV. Additionally, intracellular signaling pathways of host cells are regulated to promote the replication and proliferation of virus particles following the invasion of PEDV. For instance, it utilizes p38 MAPK and JNK signaling pathways for optimal replication (Lee et al., 2016), while NF-κB may contribute to the translocation of viral nucleic acids from the cytoplasm to the nucleus (Cao et al., 2015). Similarly, autophagy is beneficial to PEDV replication through autophagy regulatory factors and RNA interference (Guo et al., 2017) (Table 1 and Figure 2).

**TABLE 1:** Host factors and viral proteins involved in PEDV infection.

**Infection stages**	**Host factors**	**Viral protein**	**References**
Attachment and entry	Heparan sulfate	S protein	Huan et al., 2015
	pAPN	S protein	Li et al., 2007; Nam and Lee, 2010
	Sialic acid	S protein	Wrapp and McLellan, 2019
	EGFR	S protein	Yang et al., 2018
	DC-SIGN		
	DPP4		
	TMPRSS2	S protein	Shi W. et al., 2017
	MSPL	S protein	Shi W. et al., 2017
	Occludin	S protein	Luo et al., 2017
	Integrin	S protein	Li et al., 2019
Replication and transcription	hnRNPA1	N protein	Li et al., 2018
	NPM1	N protein	Shi D. et al., 2017
Assembly and release	TMPRSS2		Shirato et al., 2011
	BST2	N protein	Kong et al., 2019
	eIF3L	M protein	Wang et al., 2020
	PPID	M protein	Dong et al., 2021
	S100A11	M protein	Dong et al., 2021

## Discussion

The circulation of PEDV has caused huge economic damage to the pig industry in the world; in particular with the emergence of PEDV variant strains, an increased prevalence of 50.21–62.10% for PEDV has been witnessed in those years (Sun et al., 2012; Antas and Woźniakowski, 2019; Liu and Wang, 2021). Although progress in the understanding of PEDV including its genome, viral structure, has been made by the efforts of numerous investigations during the past years, it seems that we still stand a little far away from completely revealing the pathogenic mechanisms of PEDV. In addition, the development of effective and preventive measures like medicines and vaccines remains on the way.

Relative to extensive studies and in-depth information on other coronaviruses like SARS-CoV, host–PEDV interactions just receive increased attention recently. One typical example is that PEDV targets intestinal epithelial cells (IECs) in the intestinal villi of pigs, while the most common *in vitro* cell culture system used to study PEDV does not derive from IECs of pigs. The development of new pig IEC-derived cell lines will undoubtedly provide an alternative, more physiologically relevant model for future studies of PEDV–host interactions.

Porcine epidemic diarrhea virus whole inactivated and attenuated virus vaccines have played an effective role in the prevention and control of PED (Kweon et al., 1999; Song et al., 2007). Besides this, PEDV genetic engineering vaccines based on the S protein which can induce the body to produce neutralizing antibodies also display broad prospects. As the most ideal vaccine for oral immunization, transgenic plant vaccine similarly has great development space (Kang et al., 2006). The numerous properties of nanoparticle, including possessing immunoadjuvant activity, generating natural immune response of antigens, easily reaching antigen-presenting cells to regulate the immune response through a variety of ways, targeting to present antigen, and releasing slowly, allow it to be an ideal candidate for preparing new vaccines against the occurrence of PED (Smith et al., 2013; Tong et al., 2020). Notably, although vaccine immunization is an effective way to prevent and control PED, it often cannot solve all of the issues for PEDV-infected pigs. Therefore, some therapeutic drugs are urgently needed as well. In this regard, validly inhibitory effects of IFN-L on the proliferation of PEDV in pig intestinal epithelial cells, together with better action than type I interferon, empower it to receive more attention (Li L. et al., 2017). Likewise, a PEDV-specific yolk antibody has also been reported to increase the survival rate of infected piglets (Lee D. H. et al., 2015). Besides these, the prokaryotic expression of a single-stranded variable region of the PEDV monoclonal antibody or small peptides identified by phage screening, which is able to bind to the PEDV receptor, can also prevent PEDV from invading host cells (Meng et al., 2014). Additionally, the key role of proteases in virus release from the cell surface and in PEDV infection enhancement render them to be important drug targets. Therefore, protease inhibitors may also be good candidates for developing anti-PEDV compounds to fight this infectious disease (Shirato et al., 2011). Similarly, some Chinese herbal extracts, such as quercetin and ginkgo peel extract, can effectively suppress the infection process of PEDV *in vitro* (Choi et al., 2009; Song et al., 2011; Lee J. H. et al., 2015; Tong et al., 2020). Interestingly, the anti-PEDV effect of quercetin was found to be independent of its HSPA1 inhibitor activity and lacking of influence on the process of virus adsorption and invasion while relying on its inhibition of the PEDV 3C-like protease. Quercetin might bind to the active site of the PEDV 3C-like protease; the binding affinity between quercetin and PEDV 3CLpro was also verified by surface plasmon resonance (SPR) (Li et al., 2020). Likewise, the anti-PEDV effect of polysaccharides in ginkgo biloba pericarp has also been described (Lee J. H. et al., 2015). Additionally, some drugs were proved to have anti-PEDV activity, while they were not tested in clinical treatment due to the cost and safety. Therefore, further understanding of PEDV pathogenetic mechanisms and improved production of druggable agents would contribute to the development of therapeutics for this virus.

Viruses are obligate intracellular parasites that are limited by the ability of their genomes. Accordingly, all viruses evolve the capability to hijack host factors for their own replication. Meanwhile, host cells also develop complex signaling networks to detect, control, and eradicate invading viruses, although these antiviral pathways are often circumvented, suppressed, or disrupted by various viral anti-mechanisms. Thus, virus–host interactions represent an ongoing evolutionary arm race to perfection at the molecular and cellular levels. Therefore, it is of far-reaching significance to deeply study the pathogenesis of PEDV and identify PEDV receptors and related host factors, thereby providing more druggable targets for the prevention and treatment of PEDV.

## Author Contributions

YH performed the literature study, drafted the structural design of the review, and wrote the manuscript. XX, LY, and AW contributed intellectually with the critical revision of the manuscript. All authors have read and approved the final manuscript.

## Conflict of Interest

AW is employed by PCB Biotechnology, LLC. The remaining authors declare that the research was conducted in the absence of any commercial or financial relationships that could be construed as a potential conflict of interest.

## Publisher’s Note

All claims expressed in this article are solely those of the authors and do not necessarily represent those of their affiliated organizations, or those of the publisher, the editors and the reviewers. Any product that may be evaluated in this article, or claim that may be made by its manufacturer, is not guaranteed or endorsed by the publisher.
